# Exploring the Link Between Interoception and Symptom Severity in Premature Ventricular Contractions

**DOI:** 10.3390/jcm13247756

**Published:** 2024-12-19

**Authors:** Alena S. Limonova, Irina A. Minenko, Anastasia A. Sukmanova, Vladimir A. Kutsenko, Sofya P. Kulikova, Maria A. Nazarova, Karapet V. Davtyan, Oxana M. Drapkina, Alexandra I. Ershova

**Affiliations:** 1 National Medical Research Center for Therapy and Preventive Medicine, Ministry of Healthcare of the Russian Federation, 101000 Moscow, Russia; anastasiya.sukmanova@gmail.com (A.A.S.); alersh@mail.ru (A.I.E.); 2 Department of Psychology, National Research University Higher School of Economics, 101000 Moscow, Russia; 3 Center for Cognitive Neuroscience, National Research University Higher School of Economics, 614070 Perm, Russia; spkulikova@hse.ru; 4 Center for Cognition and Decision Making, Institute for Cognitive Neuroscience, National Research University Higher School of Economics, 101000 Moscow, Russia

**Keywords:** premature ventricular contractions, symptoms, cardiac interoception, interoceptive accuracy, mental tracking task, heartbeat detection task, heartbeat-evoked potentials, electroencephalography

## Abstract

**Background/Objectives**: The physiological basis underlying symptomatic versus asymptomatic premature ventricular contractions (PVCs) remains poorly understood. However, symptomatic PVCs can significantly impair quality of life. In patients without structural heart disease, symptom intensity is crucial for guiding management strategies and determining the need for medical or surgical intervention. In this study, we aimed, for the first time, to examine the associations between PVC symptoms and cardiac interoception. **Methods**: This study included 34 participants with PVCs (20 women; median age = 42 years; 17 participants had asymptomatic PVCs) without concomitant disorders. Interoception was assessed through interoceptive accuracy (IA) probed by two behavioral tests—mental tracking (MT) and heartbeat detection (HBD)—and the neurophysiological marker of cardiac interoception, the heartbeat-evoked potentials (HEPs). Symptom intensity scores reported by patients served as the response variable in the regression analysis, with IA and HEP as predictors. Other factors such as sex, age, percent of body fat, trait anxiety, and alexithymia were added to the models as confounding variables. **Results**: IA_MT_ was significantly higher in patients with symptomatic PVCs. IA_MT_ and HEP modulation for the HBD task were associated with symptom intensity. A combined regression model incorporating both metrics showed the highest predictive accuracy for symptom severity. Adding confounding variables improved model quality (lower AIC); however, only the male sex emerged as a significant negative predictor for symptom intensity. **Conclusions**: Our findings confirm a significant association between interoception and PVC symptom severity. Integrating behavioral and neurophysiological interoception measures enhances symptom prediction accuracy, suggesting new ways to develop diagnostic and non-invasive treatment strategies targeting interoception in PVC management.

## 1. Introduction

Interoception refers to the processes of the perception, integration, and regulation of internal bodily signals [1]. A common approach to testing an interoceptive process is to assess cardiac interoception (or cardioception). Cardiac interoceptive accuracy (IA) can be probed in behavioral tests, which are various modifications of heartbeat counting/detection tasks. However, these tests have certain limitations [2]; therefore, a neurophysiological measure of interoceptive processing, heartbeat-evoked potentials (HEPs), is of particular interest [3]. Interoception is gaining increasing attention in various fields of research, as its dysfunction may be involved in the expression of various disorders [4]. In addition, the role of interoception in symptom experience has recently been discussed. Studies summarized in the systematic review by Locatelli et al. [5] showed conflicting results regarding the relationship between IA and symptom severity. However, data on interoception and cardiac symptom severity are limited. In a recent study by Lee et al. [6], findings indicated that interoceptive awareness, measured using the Multidimensional Assessment of Interoceptive Awareness questionnaire, was linked to improved self-care management of symptoms in patients with cardiovascular diseases. Conversely, tendencies to ignore or distract oneself from discomfort were associated with poorer self-care practices. The authors suggest that interoception could serve as a valuable target for interventions aimed at improving self-care management behaviors in patients with cardiovascular disease. However, IA is only partially related to interoceptive awareness [7]. Further investigations are required to establish the associations between IA and cardiological symptoms.

One of the most common cardiac symptoms is palpitations, and its possible relationship with cardioception has recently been discussed [8]. Premature ventricular contractions (PVCs) are a common cause of palpitations, while in asymptomatic individuals, they could also be detected as an accidental finding. On the one hand, arrhythmias may be asymptomatic [9,10]. On the other hand, the feeling of palpitations may not be caused by actual heart rhythm disturbances, with a psychiatric or somatoform disorder being an underlying cause [11,12,13]. In case of the absence of cardiopulmonary etiology, PVCs are often benign. However, they can cause symptoms that significantly impair quality of life [14]. Thus, for benign PVCs, the clinical decision about the treatment strategy depends on the severity of the symptoms. In asymptomatic individuals, no treatment may be required. Nevertheless, for patients who experience palpitations that drastically affect their quality of life, antiarrhythmic drugs or even surgical treatment (ablation) may be used [15]. These treatments, though, only aim to improve quality of life but do not affect the risk of future cardiovascular events and may have side effects.

Currently, only a limited number of studies investigated cardiac interoception in palpitation patients, demonstrating higher IA in behavioral tests compared to healthy control subjects [16,17]. However, none of these studies assessed interoception in palpitation patients using the neurophysiological marker of interoception, specifically the HEP (heartbeat-evoked potential) amplitude. Moreover, neither neurophysiological nor behavioral approaches have been used together to evaluate interoception in patients with PVCs. As noted by the authors of a recent systematic review, the results of existing studies may not be generalizable beyond populations with neurodivergent chronic conditions. They emphasize the need for further research to explore the role of interoception in the symptom experience of various physical, non-communicable chronic conditions [5]. Additionally, many studies that have explored the relationship between interoception and symptoms have relied on either questionnaires or a single behavioral test for IA assessments [5]. These methods assess different dimensions of interoception [7], which complicates the comparison of findings across studies investigating the link between interoception and symptoms. Furthermore, the use of only one behavioral test for IA assessment limits the generalizability of the findings, as each test has its own limitations [2].

We suggest that understanding the mechanisms underlying the experience of symptoms in patients with benign PVCs would pave the way for the development of new therapeutic approaches.

In our study, we proposed that the intensity of symptoms of PVCs may be associated with interoception. To test this hypothesis, we explored interoception in patients with symptomatic and asymptomatic PVCs using two approaches: (1) behavioral: we evaluated IA in two commonly used behavioral tasks: mental tracking (MT) [18] and heartbeat detection (HBD) [19]; (2) neurophysiological: we studied the neural marker of cardiac interoceptive processing [20]—the HEP amplitude and its modulation (ΔHEP). Studies have demonstrated that disturbances in interoceptive signaling are linked to the regulation of emotions and stress [21], contributing to the development of various mental health conditions, including anxiety, depression, and somatic symptom disorders [22]. Moreover, interoceptive training has been shown to improve IA, reduce anxiety, and alleviate somatic symptoms. These effects are associated with enhanced neural activity in the anterior insular cortex [23]. The insula cortex is involved in interoceptive [24] and emotional processing [25] and is also a key component of the central autonomous system network [26,27]. At the same time, stress and imbalance in autonomic regulation are among predisposing factors for PVCs [28]. Considering the research mentioned above, to explore the potential contributions of anxiety and alexithymic traits to symptom severity in patients with PVCs, we administered the State-Trait Anxiety Inventory–Trait Inventory [29] and the Toronto Alexithymia Scale [30] questionnaires.

## 2. Materials and Methods

### 2.1. Participants

This study included 34 participants (20 females; Me [Q1; Q3] = 42 [38.25; 44.75] years old; body mass index (BMI) Me [Q1; Q3] = 24.4 [21.95; 26.65]; 17 participants had asymptomatic PVCs) who were either seeking medical care or undergoing a routine health check-up. All participants provided written informed consent, approved by the local Ethics Committee at the National Medical Research Center for Therapy and Preventive Medicine, Moscow, Russian Federation. This study adhered to the principles outlined in the Declaration of Helsinki. We calculated the sample size required to detect a within-group difference between conditions (power of 0.8, significance level of 0.05) using G*Power (version 3.1.9.6). An effect size of d = 0.74 was used, derived from the reported difference in HEP amplitudes between resting state and perceptual task performance in high-symptom reporters among patients with medically unexplained symptoms [31]. A paired permutation *t*-test (from the family of *t*-tests accounting for mean difference) indicated that N = 17 per group is required. Due to the novelty of the study design for these clinical groups, a priori analyses to determine the required sample size for between-group comparisons were not feasible. Additionally, similar studies either reported no significant effect [31] or did not provide the necessary effect size measures [16].

The inclusion criteria were the following: 20–50 years old, with 720–20,000 PVCs during 24 h Holter monitoring. We included only young and middle-aged patients to reduce the risk of comorbidities and cognitive impairment. The upper limit of 20,000 PVCs was applied, as this corresponds to 14 PVCs per minute. Considering our HEP analysis (which excludes epochs with PVCs and three time-locked epochs, as described below), this threshold ensures that a sufficient number of epochs remains for analysis. Patients scoring > 11 on the Hospital Anxiety and Depression Scale (HADS, [32]) were excluded, as depression may affect interoception [33,34,35]. Structural heart pathology in the included participants was ruled out based on their previous medical examinations, including echocardiography, stress testing, and in some cases, cardiac magnetic resonance imaging.

To eliminate potential confounding factors in interoception measurements, stringent exclusion criteria were applied. The exclusion criteria were as follows: uncontrolled arterial hypertension (systolic blood pressure ≥ 140 mmHg and/or diastolic blood pressure ≥ 90 mmHg) based on office, home, or ambulatory monitoring; other arrhythmias observed using ECG or Holter monitoring; organic cardiac pathologies (e.g., heart hypertrophy, prior myocardial infarction, cardiomyopathies of various etiologies, unknown scarring, and congenital heart defects); obstructive sleep apnea syndrome; significant atherosclerosis (arterial stenosis ≥ 50%); neurological or psychiatric disorders; thyroid dysfunction; systemic or autoimmune diseases; significant liver, kidney, or lung pathologies; epilepsy; head trauma in the past year; use of drugs crossing the blood–brain barrier; complications from viral/infectious diseases; endocrine disorders (e.g., diabetes and obesity [>30 kg/m^2^]); and pregnancy.

### 2.2. Data Acquisition

#### 2.2.1. Symptom Score

Patients were divided into symptomatic and asymptomatic groups based on the results of a cardiologist consultation and confirmed by Holter ECG monitoring results prior to EEG administration, supplemented by patient diaries documenting their sensations during the monitoring period. Patients in whom PVCs were an incidental finding and not associated with any complaints were assigned to the asymptomatic group. In the case complaints in the diary (such as irregular heartbeat, stopping, jumping, feeling of extra or skipped heartbeats, and pounding or vigorous heartbeats) corresponded to PVCs on the ECG, patients were assigned to the symptomatic group. Participants in this group were then asked to rate the intensity of their PVC complaints on a scale of 1 to 10. Patients in the asymptomatic group were assigned a symptom score of 0. Patients in the symptomatic group were assigned a symptom score of 1, 2, or 3 for the first, second, and third tertiles of the reported symptom severity, respectively.

#### 2.2.2. Questionnaires

We used the Toronto Alexithymia Scale (TAS-20) [30] (Russian language adaptation [36]) for the alexithymia assessment to evaluate difficulties in recognizing and expressing emotional experiences and bodily sensations. The scale consists of 20 questions rated from 1 to 5 points, with higher scores indicating higher levels of various aspects of alexithymia. The total alexithymia index was calculated as the sum of three subscales: difficulties identifying feelings, difficulties describing feelings, and externally oriented thinking. To assess anxiety, we used the State-Trait Anxiety Inventory–Trait Inventory (STAI-T) [29] (Russian language adaptation by Hanin [37]). We used the trait anxiety subscale, containing 20 statements rated from 1 to 4 points.

#### 2.2.3. Electrophysiological Data

Electrophysiological data were collected using the NVX-52 EEG amplifier (Medical Computer Systems, Ltd., Moscow, Russia [MCS]) with 36 Ag/AgCl electrodes positioned on elastic EEG caps following the international 10–20 system. Channels T3 and T4 served as ipsilateral online references, following the company recommendations. During the recordings, the impedance across all channels was maintained at around 10 kΩ and never exceeded 20 kΩ. Surface electromyography (EMG) was recorded from the first dorsal interosseous muscle using a belly-tendon montage. ECG data were obtained using three pairs of electrodes in a bipolar configuration: one pair was placed on the anterior forearm and another on the chest 2 cm below the clavicles in the infraclavicular fossae [38]. Additionally, two EOG electrodes were placed on the lateral sides of the eyes. Data were sampled at a frequency of 500 Hz and filtered between 0.1 and 70 Hz with a 50 Hz notch filter.

#### 2.2.4. Other Variables

Body fat percentage, a more precise indicator of body composition than BMI, was assessed using a bioimpedance analysis (Medass, Moscow, Russia). For the maximum PVC count during 24 h Holter monitoring, we report the results from the Holter monitoring session that recorded the highest PVC count from the six months before the experiment.

### 2.3. Experimental Procedures

The participants were sitting quietly with their eyes open. At the onset, five minutes of resting EEG data were recorded, during which participants were instructed to focus on the fixation cross in the middle of the screen in front of them and to avoid moving and excessive blinking. Before each of the behavioral tests, presented in random order, participants had a training session and were instructed to focus on their internal sensations.

The MT task involved counting the heartbeats during six randomly presented time intervals (25, 30, 35, 40, 45, and 50 s) [7]. The participants had to count the number of perceived heartbeats without palpating the pulse anywhere on the body or trying to guess it.

The HBD task [39] required participants to detect the sensation of their heartbeat and indicate it by pressing a button. The test consisted of two conditions: (1) an interoceptive condition where participants had to press the “space” button when they felt a heartbeat, and (2) an exteroceptive condition where participants were required to press the button every time they heard a 1 Hz sound signal with 500 ms duration. Both conditions consisted of a 2.5 min trial and a 10 s training trial before it. Participants were instructed to report/press only perceived heartbeats, which helps to reduce the influence of estimation and better capture interoceptive ability [40,41,42], in contrast to earlier studies, where participants were asked to rely on their intuition in case they did not feel heartbeats [43,44].

### 2.4. Data Preprocessing

#### 2.4.1. Behavioral Interoceptive Accuracy (IA)

IA in the HBD task (IA_HBD_) was a metric of the mean difference (mean distance, md) between the pressing frequency and heartbeat frequency in the overlapping time windows [45,46] (Equation (1)). As demonstrated previously ([47] and personal unpublished data), this metric for the HBD task was more reliable than the other metrics (the modified Schandry index, sensitivity d). Participants who did not make any presses were assigned an IA_HBD_ of 0. The ECG recording was divided into overlapping windows of 10 s duration beginning with the R-peak. The press-to-press intervals (PP intervals) and R-peak intervals (RR intervals) were evaluated in each window. The variation was calculated as the ratio of the standard deviation of PP intervals to their mean in the window. The window was included in the analysis if the variation of PP intervals was <0.5. The formula is as follows:

md = 1 − 1/N∑ |1/PP − 1/RR|(1)

IA in the MT task (IA_MT_) was assessed using a modified Schandry index (SI) formula proposed by Garfinkel et al. [7] (Equation (2)). The SI corrects the IA_MT_ if the number of heartbeats counted by the participant (HBreport) is significantly higher than the actual number of heartbeats recorded by the ECG (HBrecord):

IA_MT_ = 1/6∑(1 − (|HBrecord − HBreport|)/((HBrecord + HBreport)/2)) (2)

An IA_MT_ and IA_HBD_ equal to 1 indicate high interoception.

#### 2.4.2. ECG, EMG Processing

The first processing step was to calculate the difference in activity between the left and right electrodes for each pair of ECG electrodes. The ECG data were then filtered between 0.5 and 45 Hz. The ECG signal from the pair of electrodes below the clavicles was used to calculate R-peaks as they were the least affected by motor activity. R-peaks and PVCs were determined semi-automatically using the MNE-Python package (version 3.6.3) [48], followed by visual inspection by a cardiologist. Both sinus rhythm peaks and PVCs were included in the IA calculation.

EMG data were used to assess the precise timing of the button press. The Teager Kaiser Energy operator was applied to EMG to detect the movement onset.

#### 2.4.3. EEG Processing

The EEG was processed using the MNE-Python package [48]. The EEG was notch-filtered at 50 Hz, and then bandpass filtered from 0.5 to 45 Hz using a zero-phase FIR filter with a Hamming window. The EEG data were cleaned from the eye movement and cardiac field artifacts (CFAs). The independent component analysis (the fastica algorithm) was used on top of the PCA components, explaining 99% of the variance. We selected no more than three eye-movement-related components and no more than two CFA components. The EEG data were then filtered between 0.5 and 20 Hz. Channels affected by motor and technical artifacts were removed and then interpolated.

#### 2.4.4. Heartbeat-Evoked Potentials (HEPs) Analysis

The EEG data were segmented into epochs 200 ms before and 600 ms after the R-peaks in the ECG, and the average amplitude over the interval from 200 to 100 ms before the R-peak was subtracted for baseline correction. HEP amplitudes for each channel were obtained from epoch-wise averaging. We excluded epochs with PVCs as well as 2 preceding it and one following it, and epochs with R-peak intervals shorter than 600 ms. The AutoReject algorithm was applied to remove or interpolate noisy epochs. After automatic artifact removal, additional visual inspection of the epochs was performed. Recordings with more than 20% of epochs removed were excluded from the analysis. Thus, out of 37 subjects, 2 were excluded from the analysis (more than 20% were removed due to frequent (838 and 820 PVCs) PVCs during recordings), and data from 1 subject were missing due to technical problems during the recording. The number of epochs included in each condition for the symptomatic and asymptomatic groups was as follows: 288 ± 79 and 272 ± 44 during rest, 144 ± 34 and 139 ± 23 during the interoceptive condition of the HBD task, 145 ± 29 and 142 ± 21 during the exteroceptive condition of the HBD task, and 219 ± 51 and 205 ± 32 during the MT task. Channels were combined into spatial ROIs as previously described [44,49]: left frontal—LF (LF: Fp1, F3, FC3, C3, F7, FT7), central frontal—CF (Fpz, Fz, FCz, Cz), right frontal—RF (Fp2, F4, FC4, C4, F8, FT8), left parietal—LP (TP7, CP3, P3, T5, P5, PO7), central occipital—CO (CPz, Pz, POz, Oz, PO3, O1, PO4, O2), and right parietal—RP (TP8, CP4, P4, T6, P6, PO8). HEP amplitudes among ROIs were obtained from the within-ROI epoch-wise averaging of the HEP amplitude in the range of 200–600 ms after the R-peak.

### 2.5. Statistical Analyses

#### 2.5.1. Analysis of Behavioral Data

The analysis was performed using the open-source R 4.3.1 environment. *p* < 0.05 was considered statistically significant. Data normality was assessed using the Shapiro–Wilk test. Between-group comparisons of IA were performed using the Wilcoxon rank-sum test for independent samples with Bonferroni correction. A two-sided Spearman’s rank correlation coefficient was used to investigate the relationships between IA_MT_ and IA_HBD_ within groups.

#### 2.5.2. HEP Statistical Analyses

HEP amplitudes were compared using a nonparametric spatiotemporal permutation test based on the Monte Carlo method. The test was performed using Python 3.11 and the MNE-Python [48]. First, HEP amplitudes were randomly assigned to groups, and t-values were calculated at each time point across channels. Next, the cluster with the largest sum of t-values was selected from the points where t-values exceeded a threshold. These steps were repeated 1000 times to form a distribution of t-values and test the null hypothesis. Based on the statistics, areas in the original data where t-values exceeded the threshold by more than 5% were selected and grouped into clusters, considering the spatial connectivity of the channels. The HEP amplitude between groups was compared for six conditions (summarized in Table 1). Within-group comparisons were performed for (1) HEP_HBD_ compared to HEP_REST_, (2) HEP_MT_ compared to HEP_REST_, and (3) HEP_HBD_ compared to exteroceptive conditions in the HBD task. The window of examination was from −200 to 600 ms.

A binary logistic regression model was individually fitted for each of the six conditions to predict the presence (coded as 1) or absence (coded as 0) of symptoms. The model was based on the averaged HEP amplitudes recorded in the ROIs for each condition over a 200–600 ms time window. The significance of the models was tested by the likelihood ratio test (LRT).

A nonparametric Wilcoxon test for dependent samples was used to compare HEP_REST_ and HEP_HBD_, HEP_REST_ and HEP_MT_, and the interoceptive and exteroceptive conditions in the HBD task in ROI ∈ {LF, CF, RF, LT, RT, CO} (Section 4.3) within groups. The Benjamini–Hochberg (BH) correction was applied to the *p*-values obtained for ROIs within each of the three conditions.

#### 2.5.3. Correlation Between HEP Amplitude and IA

We assessed the link between the behaviorally assessed IA and the neurophysiologic measure of interoception—the HEP amplitudes in these tasks (IA_HBD_ and HEP_HBD_, IA_MT_ and HEP_MT_, IA_HBD_ and ΔHEP_HBD-REST_, IA_MT_ and ΔHEP_MT-REST_, IA_HBD_, and ΔHEP_HBD-EX_). The previously described permutation test was applied for the whole sample. First, IA was shuffled between participants rather than HEP amplitudes. Second, t-statistics were calculated in steps. Initially, a two-sided Spearman’s rank correlation coefficient between HEP amplitude and IA was calculated in the randomized data from each permutation and the observed data. The coefficient was then converted to a t-statistic.

#### 2.5.4. Exploratory Regression Analysis

The analysis was performed using the open-source R 4.3.1 environment for the whole sample. Firstly, to explore how confounding variables (sex, age, percent body fat, STAI-T score, and TAS-20 total score) influenced symptom scores, we implemented Poisson Generalized Linear Models (GLMs) (Model A0). Secondly, we explored how symptom score was predicted in GLMs separately by behavioral and electrophysiological metrics (IA in tasks, HEP amplitude in ROI ∈ {LF, CF, RF, LP, CO, RT}) (simple Models A1–A5):

A1. Symptom score ~ IA_HBD_
A2. Symptom score ~ IA_MT_
A3. Symptom score ~ ΔHEP_MT-REST_ in ROI ∈ {LF, CF, RF, LP, CO, RT}
A4. Symptom score ~ ΔHEP_HBD-REST_ in ROI ∈ {LF, CF, RF, LP, CO, RP}
A5. Symptom score ~ ΔHEP_HBD-EX_ in ROI ∈ {LF, CF, RF, LP, CO, RP}

Then, to explore how confounding variables influenced the predictions, for models 1–5, we implemented multiple models adding confounding variables (Models B1–B5). Covariates in each model were tested for the lack of multicollinearity (the variance inflation factor (VIF) did not exceed the threshold of 5). For simple and multiple models 3–5, we used a BH correction on the *p*-values obtained for ROIs within each of the three conditions.

Finally, we performed a data-driven analysis using behavioral and electrophysiological interoception metrics that yielded significant associations with the symptom scores (Models A6–A7 and B6–B7). We used the Akaike information criterion (AIC) to estimate the relative quality of GLMs.

To investigate whether the covariates moderate the relationship between the interoception metrics and symptom severity, we performed moderation analyses. We fitted models Symptom score ~ interoception + covariate + interoception·sex, where interoception—one of the predictors (IA/HEP) of final models, covariate—covariate which demonstrated significant association in the multiple models of regression analyses.

## 3. Results

### 3.1. Sample Characteristics

Table 2 presents the clinical parameters of the study groups. The groups were comparable in age, but the asymptomatic group had significantly more males, a significantly higher BMI (but within the normal range for both groups), and the number of PVCs during the 24 h Holter. The groups did not differ in anxiety and alexithymia. In total, seven (41%) participants from the symptom group had symptom severity < 6 and were assigned a symptom score of one, four (24%) participants had a severity between 6 and 7.83 and a symptom score of two, and six (35%) participants had a severity > 7.83 and a symptom score of three.

### 3.2. Interoceptive Accuracy (IA)

**Figure 1:**
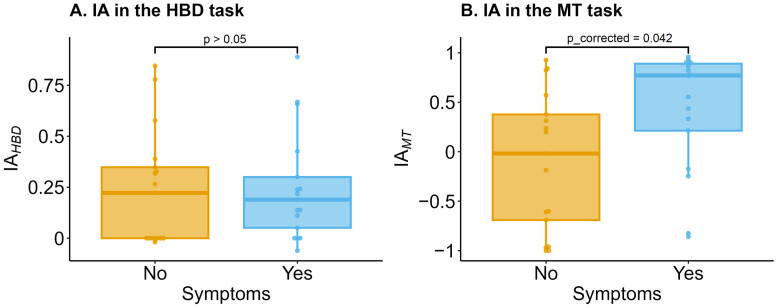
Comparison of (**A**) interoceptive accuracy (IA) in the heartbeat detection task (IA_HBD_) and (**B**) IA in the mental tracking task (IA_MT_) between symptomatic (symptoms yes) and asymptomatic groups (symptoms no). Horizontal lines represent the medians, boxes span the interquartile range (IQR), and whiskers extend to 1.5 × IQR. Points represent individual data. Wilcoxon criterion with Bonferroni correction was used.

IA_HBD_ did not differ between groups (W = 138.5, *p* = 0.84). IA_MT_ in the symptomatic group (*n* = 17, Me [Q1; Q3]) = 0.77 [0.21; 0.89]) was significantly higher (W = 78, *p* = 0.021, after applying the Bonferroni correction p_corrected_ = 0.042) than in the asymptomatic group (*n* = 17, Me [Q1; Q3]) = 0.22 [0; 0.35]) (Figure 1).

**Figure 2:**
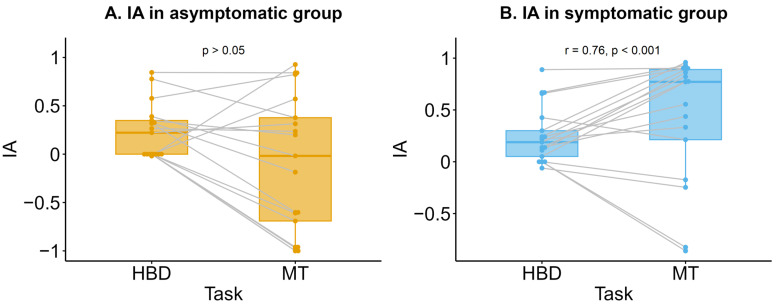
The Spearman correlation between interoceptive accuracy (IA) in the heartbeat detection task (HBD task) and in the mental tracking task (MT task) within groups. (**A**) Asymptomatic group. (**B**) Symptomatic group. Horizontal lines represent the medians, boxes span the interquartile range (IQR), and whiskers extend to 1.5 × IQR. Points represent individual data. Lines connect the IA values for two tests of each patient.

The Spearman correlation between IA_HBD_ and IA_MT_ was significant for the symptomatic group (r = 0.76, *p* < 0.001) in contrast to the asymptomatic group (r = 0.42, *p* = 0.09) (Figure 2).

### 3.3. Heartbeat-Evoked Potentials (HEPs) Results

**Figure 3:**
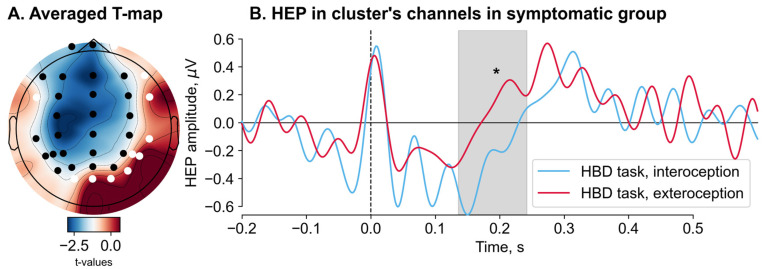
Heartbeat-evoked potentials (HEPs) amplitude comparison between the interoceptive and exteroceptive conditions of the heartbeat detection task (HBD task) in the symptomatic group (nonparametric permutation paired *t*-test). HEP was averaged over a window of −200 to 600 ms relative to the onset of the R-peak in the ECG. The results identify the cluster, defined as the channels and time periods, where HEP was significantly different between conditions. (**A**) Topographic representation of the obtained t-statistics (T-map) for each channel over the head. The map shows mean statistics for the significant time period when HEP differed. Channels included in the cluster are colored black, while the remaining channels are colored white. (**B**) HEP amplitudes during the interoceptive and exteroceptive conditions in the HBD task. HEP was averaged across the channels comprising the significant cluster (black channels in panel **A**). The significant time range is highlighted in gray. * Monte Carlo *p* = 0.029.

We found no significant difference in the HEP amplitude during all six conditions between the symptomatic and asymptomatic groups. A significant effect of condition was observed in the symptomatic group when comparing the HEP amplitudes recorded during the interoceptive and exteroceptive conditions in the HBD task (Monte Carlo *p* = 0.029) (Figure 3). A significant cluster, spanning from 136 to 244 ms, included the following channels: Fp1, Fpz, F7, F3, Fz, F4, FC3, FCz, FC4, C3, Cz, C4, TP7, CP3, CPz, CP4, T5, P3, Pz, P4, P5, PO3, POz, PO4, and PO7. Cluster-averaged HEP amplitudes for the interoception (M = −0.39, SD = 0.4) were significantly less than for exteroception (M = 0.03, SD = 0.54). After restricting the time window from 200 to 600 ms, this result became nonsignificant.

**Figure 4:**
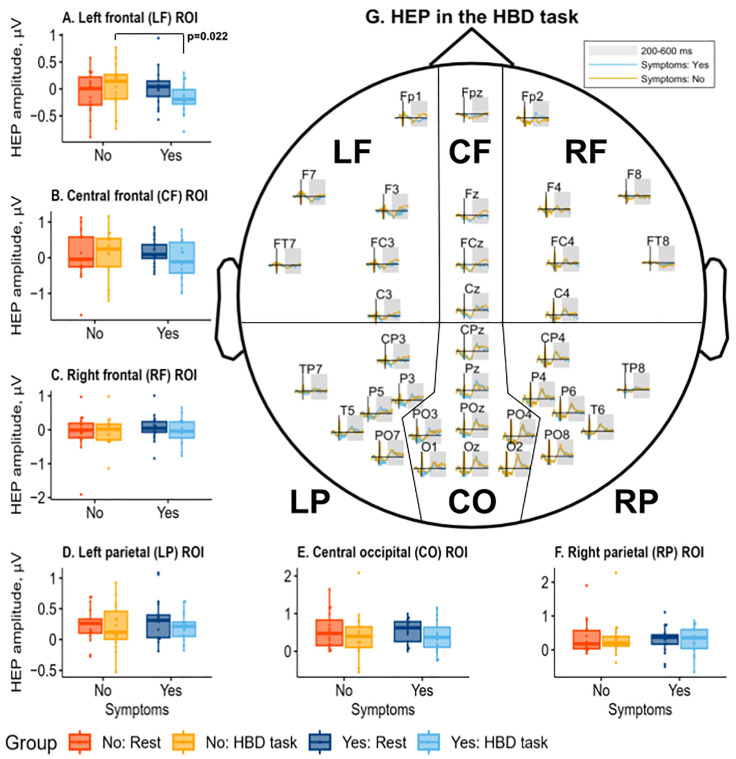
(**A**–**F**) Mean amplitudes of heartbeat-evoked potentials (HEPs) averaged across channels in a window of 200–600 ms relative to the onset of the R-peak. Channels were grouped into six predefined regions of interest (ROIs): (**A**) left frontal (LF), (**B**) central frontal (CF), (**C**) right frontal (RF), (**D**) left parietal (LP), (**E**) central occipital (CO), and (**F**) right parietal (RP). HEP amplitudes were grouped based on (1) the presence of symptoms of premature ventricular contractions (PVCs) in patients and (2) the conditions during which HEPs were recorded (Rest and during heartbeat detection task (HBD task)). Boxplot colors correspond to the conditions and groups: asymptomatic PVCs in the HBD task (light orange), asymptomatic PVCs during rest (dark orange), symptomatic PVCs in the HBD task (light blue), and symptomatic PVCs during rest (dark blue). Horizontal lines represent the medians, boxes span the interquartile range (IQR), and whiskers extend to 1.5 × IQR. Points represent individual data. The statistical significance of HEP in the HBD task within the LF ROI for predicting the presence of symptoms is indicated in panel (**A**). (**G**) Topographic representation of HEP amplitudes over the head during the interoception condition of the HBD task. Grand averages of HEP amplitudes over the 200–600 ms window relative to the R-peak are shown in each channel separately for symptomatic and asymptomatic groups (indicated by color). The 200–600 ms window is shaded in gray.

The amplitude of HEP in ROIs did not differ between conditions in the asymptomatic group. HEP_REST_ in CF ROI (M = 0.17, SD = 0.37) was higher (W = 33, *p* = 0.04) than HEP_HBD_ (M = −0.07, SD = 0.55) in the symptomatic group; however, the result did not survive the BH correction (p_BH_ = 0.12) (Figure 4).

**Figure 5:**
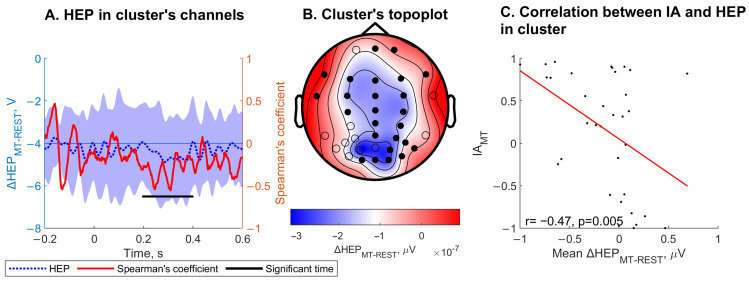
Spearman’s rank correlation coefficient between heartbeat-evoked potentials modulation in the mental tracking (MT) task compared to rest (ΔHEP_MT-REST_) and interoceptive accuracy for this task (IA_MT_) for all patients. HEP amplitudes were averaged over a window of −200 to 600 ms relative to the onset of the R-peak. Permutation *t*-test on the correlation data was conducted. The results identify the cluster, defined as the channels and the time period, where HEP was significantly correlated with IA. (**A**) The dotted blue line represents the ΔHEP_MT-REST_ averaged over the significant cluster channels. The shaded blue area represents the standard deviation. The black line indicates the significant cluster period. (**B**) Topographic representation of mean ΔHEP_MT-REST_ during the significant cluster period in each channel over the head. Channels included in the cluster are highlighted in black. (**C**) Scatter plot of IA_MT_ and ΔHEP_MT-REST_. ΔHEP_MT-REST_ represents the mean amplitude in the significant cluster channels and during the significant cluster period.

ΔHEP_MT-REST_ averaged within channels and over cluster time was significantly correlated with IA_MT_ (r = 0.47, *p* = 0.005) within one cluster (Monte Carlo cluster *p* = 0.005 (Figure 5)). The cluster spanning channels included Fpz, Fp2, F7, F3, Fz, F4, F8, FT7, FC3, FCz, FC4, C3, Cz, C4, CPz, CP4, Pz, P4, T6, PO4, P6, O1, Oz, O2, and PO8 and the time period ranged from 198 to 398 ms. ΔHEP_MT-REST_ was significantly correlated with IA_MT_. HEP_HBD_, HEP_MT_, ΔHEP_HBD-REST_, and ΔHEP_HBD-EX_ did not correlate with IA for the corresponding task.

HEP_HBD_ in LF ROI was a significant negative predictor of symptom presence (*n* = 34, β = −7.35, *p* = 0.022), and the LRT in the logistic regression model showed significance (*p* = 0.047) (Figure 4). HEP_MT_ in RP ROIs was a significant negative predictor of symptom presence (β = −7.2, *p* = 0.035), but the LRT in the logistic regression model showed non-significance (*p* = 0.21). HEP_REST_, ΔHEP_HBD-REST_, ΔHEP_MT-REST,_ and ΔHEP_HBD-EX_ were not significant predictors of symptom presence.

### 3.4. Regression Analyses

Male sex in Model A0 emerged as a significant factor for lower symptom scores (β = −1.22, *p* = 0.028). IA_MT_, but not IA_HBD_, showed a significant positive effect on the symptom score (β = 0.82, *p* = 0.01) in a simple model, which was sustained when adding clinical parameters (β = 0.77, *p* = 0.025) in a multiple model.

ΔHEP_HBD-REST_ in LF (*p* = 0.017, p_BH_ = 0.037) and CF (*p* = 0.016, p_BH_ = 0.033) ROIs and ΔHEP_HBD-EX_ in LF (*p* = 0.025, p_BH_ = 0.037) and CF (*p* = 0.022, p_BH_ = 0.033) ROIs were significant negative predictors of symptom score in a simple model. The result for ΔHEP_HBD-REST_ in LP (*p* = 0.037, p_BH_ = 0.112) ROIs did not survive the BH correction. Adding clinical parameters to the model made the effect of ΔHEP_HBD-EX_ on the symptom score nonsignificant in LF and CF ROIs and reduced to tendency the effect of ΔHEP_HBD-REST_ in CF (*p* = 0.019, p_BH_ = 0.058) ROIs. A trend toward a significant association for ΔHEP_HBD-REST_ in LP (*p* = 0.019, p_BH_ = 0.058) and CO (*p* = 0.018, p_BH_ = 0.055) was observed only in the multiple models. ΔHEP_MT_ was not associated with the symptom score (Table 3).

The final models included ΔHEP_HBD-REST_ in ROI ∈ {LF, CF, LP} and IA_MT_ and ΔHEP_HBD-EX_ in ROI ∈ {LF, CF} and IA_MT_ as predictors of the interoception. The best model based on the AIC was model B6, where ΔHEP_CONDITION_ is ΔHEP_HBD-REST_ in LP ROIs (Table 4). The interaction between interoception (IA or HEP) and sex was not significant, indicating that the relationship between interoception and symptom score was not moderated by sex.

## 4. Discussion

We hypothesized that the intensity of symptoms in PVCs may be associated with interoception. To test this hypothesis, we compared the IA in two behavioral tests (MT and HBD) and a neurophysiological marker of cardiac interoceptive processing—the HEP—between symptomatic and asymptomatic individuals with PVCs. Interestingly, patients in the asymptomatic group had significantly higher PVCs both during the experimental procedure and during 24 h Holter monitoring. This precludes discussing the results regarding symptom severity just in the context of the total number of PVCs. An important aspect of our HEP analysis is excluding epochs with PVCs and time-locked ones (two before and one after) to avoid their influence on HEPs. This approach ensures that only basal HEP, which can be considered a marker of interoception as a personal trait, is evaluated.

Currently, there are no broadly accepted theories that explain symptom intensity in cardiac diseases. However, some models address medically unexplained symptoms. One of these models is a perception-filter model [50]. Schulz et al. [31] tested the assumptions of this model to investigate alterations in the neurophysiological processing of the interoceptive signals in individuals with medically unexplained symptoms (not exteroceptive, as in previous studies [51,52], on which the perception-filter model was mainly based). The authors tested all three levels of this model: afferent bodily signals (evaluated via heart rate and heart rate variability), filter system activity (evaluated via ΔHEP), and perception (evaluated via IA in the behavioral tests—the MT and heartbeat discrimination tasks). The authors hypothesized that high-symptom reporters would have (1) a higher heart rate and relative sympathetic tone, (2) higher IA than low-symptom reporters, and (3) lower filter activity, indicated by a lower ΔHEP. Our results are in accordance with their hypothesis.

### 4.1. Interoceptive Accuracy (IA) and Symptoms

Hypervigilance models predict higher IA, which aligns with our findings of greater IA_MT_ in symptomatic patients [53]. It could be argued, however, that the higher IA_MT_ in symptomatic individuals does not necessarily reflect true heartbeat perception. Instead, it may stem from symptomatic individuals’ belief that they perceive their heartbeats, leading them to report a higher number, which incidentally matches the actual heartbeat count. Indeed, one of the limitations of the MT task is its inability to distinguish between true sensitivity and response bias [54]. To explore this further, we calculated the modified Schandry index for HBD [45,46,47], where a higher score indicates more frequent tapping during HBD. No difference in this index was found between groups. This result could be extrapolated to some extent to the results of MT in the sense that patients from the symptomatic group are not characterized by reporting higher numbers independent of actual perception. Additionally, IA_MT_ and IA_HBD_ correlated only in the group of symptomatic individuals, consistent with findings by Kormendi et al. [55]. Furthermore, in contrast to the findings of Schulz et al., 2020 [31], our results demonstrated a correlation between IA and ΔHEP, but only for MT, not HBD tasks. This suggests that increased attentional resources directed toward bodily sensations may enhance perceptual accuracy. These findings further support the fact that the difference in IA_MT_ between symptomatic and asymptomatic patients with PVCs reflects actual differences in interoception. Our results of higher perception are in line with a previous study by Petersen et al. [56], who found that individuals reporting more frequent symptoms and higher levels of negative affect also showed greater accuracy in an interoceptive classification task, where they were asked to discriminate between various respiratory stimuli.

### 4.2. Heartbeat-Evoked Potentials (HEPs) and Symptoms

Attention to heartbeats has previously been shown to modulate HEP amplitude [57,58,59]. We initially hypothesized that symptomatic individuals would have a higher HEP_REST_, an indicator of hypervigilance to internal signals, consistent with cognitive–behavioral models (for a review, see [53,60]). In this case, due to this permanent heightened attention to the body, ΔHEP during the interoceptive test should be lower than in asymptomatic individuals. However, we found no differences in HEP_REST_ between the two groups. This may be due to the small sample size, as the regression analysis did capture the association between ΔHEP and symptom severity.

Within-group analyses of ΔHEP showed a significant difference between exteroceptive and interoceptive tasks in the time window from 136 ms to 244 ms for the symptomatic group, with more negative HEPs observed in the interoceptive condition. However, when the analyses were restricted to the 200–600 ms time window, this result was no longer significant. While some studies selected a time window of 200 ms or more after the R-peak to avoid contamination of the HEP with cardiac artifacts [21,39,57], other studies did not impose such limitations after removing cardiac field artifacts [43,58,61]. These studies demonstrated HEP differences beginning before 200 ms, which aligns with our findings.

Results regarding the direction (positive or negative) of HEP deflection during cardiac interoceptive tasks vary across studies. Our results indicated that a lower ΔHEP in the HBD task (both ΔHEP_HBD-REST_ and ΔHEP_HBD-EX_) was associated with higher symptom intensity. In a study by Couto et al. [43], a positive deflection of ΔHEP_HBD-REST_, but not of ΔHEP_HBD-EX_, was observed in control subjects. In contrast, other studies demonstrated both negative ΔHEP_HBD-REST_ [61] and ΔHEP_HBD-EX_ deflection [61,62] in control subjects as well as in patients with hypertension [39] and obsessive–compulsive disorder [61], but not in patients with panic disorder [61]. Additionally, the study by Leopold et al. [63] showed no difference in HEP_HBD_ compared to the exteroceptive condition. For the MT task, a higher HEP_MT_ was observed compared to both the exteroceptive condition [59] and HEP_REST_ in control subjects [21], although this was not the case for patients with depersonalization disorders [21]. Furthermore, a higher HEP_MT_ was reported in high-symptom reporters but not in low-symptom reporters [31]. In another study, participants did not perform any cardiac interoceptive tasks but were instructed to focus their attention either on their own heartbeat or on white noise emitted through headphones. HEPs were significantly higher during attention to the heartbeat [57].

The insular cortex, prefrontal cortex, and left somatosensory cortex [24,64] as well as the frontal temporal cortex [65] were described as one of the sources of HEP generation. Previous studies in the sensor domain showed that there is an effect of attention to heartbeat pronounced in the modulation of HEP in frontal central regions [3], as well as an effect of AF presence in patients characterized by the modulation of HEP in the frontal temporal regions [66]. The left lateralization of insular involvement during the HBD task was demonstrated by Fittiapldi et al. [47]. We found modulation in the left frontal, fronto-central, and left temporal regions, which is in line with previous studies. We hypothesize that HEP modulation in these regions may indicate a role of interoception in the formation of symptoms associated with cardiovascular pathology.

Our findings on HEP modulation can be interpreted within the framework of the predictive processing model. This model suggests that the brain continuously formulates predictions about internal and external states while minimizing prediction errors by integrating sensory input with prior expectations [67,68]. Prediction errors can be reduced through several mechanisms: by updating generative models of interoceptive states, by activating autonomic reflexes to modify interoceptive signals through active inference, or by engaging in behaviors that alter external conditions influencing internal homeostasis [69]. In healthy individuals, prediction errors are typically attenuated, but a failure in this mechanism could lead to hypervigilance toward incoming interoceptive signals. This aligns with our findings, which show increased IA in the symptomatic group. HEP modulation is thought to reflect the brain’s capacity to adapt predictions about bodily states during tasks that involve heartbeat awareness [70,71]. No prior research has specifically explored the relationship between HEP modulation and symptom severity in patients with cardiological disorders. However, assuming the cross-nosological role of interoception in symptom perception, the findings from related studies are relevant. Petzschner et al. [57] identified a negative association between HEP modulation and the supradiaphragmatic reactivity subscale of the Body Perception Questionnaire. High scores on this subscale reflect the increased reactivity of supradiaphragmatic organs, such as the heart, based on the perceived frequency of potential warning signals from these organs. This finding aligns with our results, which demonstrated that HEP modulation negatively predicts symptom severity. One possible explanation is that an increased or heightened perception of interoceptive signals may arise from an inability to downregulate the precision or salience of these signals in situations where bodily attention is unnecessary. Furthermore, Pang et al. [72] proposed that HEP amplitudes are influenced not only by conscious but also by subconscious attention to interoceptive signals. Their study demonstrated that HEP modulation occurred even when participants were not instructed to focus on their heartbeats, with HEP being recorded during eyes-closed (interoceptive) and eyes-open (exteroceptive) states. They also observed differences in HEP modulation between healthy individuals and patients with generalized anxiety disorder. Notably, in their study, the HEP amplitude during interoceptive conditions was significantly correlated with the severity of symptoms (anxiety) in patients. This suggests that dysfunction in HEP modulation could be linked to excessive interoceptive processing, characterized by aberrant attention to bodily sensations, which, in turn, leads to their conscious perception.

### 4.3. Multimodality of Symptom Intensity

The multimodal nature of interoception is known to be influenced by factors such as sex and age [73,74]. Studies on the contribution of clinical factors to cardiac disease symptomatology have mainly focused on patients with atrial fibrillation. According to them, it can be concluded that female sex and young age were associated with symptomatic AF [75,76,77], while male sex and older age were associated with asymptomatic atrial fibrillation [78,79]. In studies with gut interoception, female sex was also associated with gastrointestinal symptom severity [80]. Similarly, patients with palpitation caused by an awareness of sinus rhythm were significantly more likely to be women than those with palpitation due to arrhythmias [81]. The distribution of sex in our groups is consistent with this (with female preponderance in the symptomatic group), and regression analyses showed that male sex predicted a low symptom score. However, there is a study showing that patients who underestimated the length and frequency of atrial fibrillation episodes could be predicted by female sex [82] and a study where older patients had better matching of symptoms with ECG activity [10]. The absence of age-related effects on symptom scores in our study aligns with findings from a previous study, which reported that age did not predict symptoms in asymptomatic patients with atrial fibrillation, atrial flutter, or atrial tachycardia following ablation [9]. However, while prior research has demonstrated an association between symptom severity and age in atrial fibrillation patients, even within a narrower age range [77], the lack of age-related influence in our study may be explained by the intentionally restricted age range of participants. This selection was designed to minimize the impact of chronic conditions commonly observed in older individuals.

Rouse et al. (1988) showed a negative relationship between IA and BMI and the percentage of fat mass in healthy individuals [83]. Patients with a higher BMI also demonstrated interoceptive deficits [84]. Studies on other clinical groups with comorbid overweight and obesity have shown a direct association between increased BMI and greater symptom severity. For instance, a higher BMI was linked to more severe symptoms in women with fibromyalgia syndrome, as measured by the Fibromyalgia Impact Questionnaire (FIQ-R) [85]. Similarly, in patients with atrial fibrillation, elevated BMI was associated with higher symptom burden scores, assessed using the Toronto Atrial Fibrillation Severity Scale (AFSS) [86]. Additionally, in patients with metabolic syndrome, body fat percentage was correlated with increased severity of anxiety and depressive symptoms [87]. In our study, the lack of influence of body composition on symptom scores can be explained by the fact that (1) there was no variability in body fat percentage in the non-obese sample, which did not allow for testing associations, and (2) body composition is not involved in the formation of PVC symptomatology.

Alexithymia, defined as difficulties in identifying and describing one’s own feelings, is characterized by impaired interoception [88], which, in turn, is related to emotional processing [89]. Previous research has demonstrated a higher prevalence of alexithymia in patients with medically unexplained symptoms, with a correlation between alexithymia and symptom severity [90]. Therefore, we hypothesized that the presence or absence of symptoms of PVCs may be associated with alexithymia. However, multiple regression models identified no associations between alexithymia and symptom intensity as the response variable. Although inverse associations between IA_MT_ and alexithymic traits in a group of healthy subjects have been demonstrated [88,91], recent meta-analyses by Desmedt [73] have revealed no significant associations between IA_MT_ and trait anxiety or alexithymia. The authors hypothesized that this may be due to the inability of the IA_MT_ to adequately capture individual differences in interoception. Indeed, the existing literature on the relationship between interoception and alexithymia is inconclusive, with findings varying depending on the methodological approach employed. In the study by Flasbeck et al. [92], there was a positive correlation between HEP amplitudes over frontal electrodes and alexithymia for the entire study sample (control subjects and patients with borderline personality disorder), but not within the aforementioned groups. This may explain the absence of associations between alexithymia and symptom severity in our study because participants did not have any psychological or psychiatric disorders and exhibited low alexithymia. Consequently, the TAS-20 score may not have been sufficiently sensitive to detect subtle differences in emotional processing.

Palser et al. [93] demonstrated that the combination of altered interoceptive processing and alexithymia is linked to a higher risk of developing anxiety disorders compared to altered interoception alone. Notably, palpitations are among the common symptoms reported by patients with anxiety disorders [13]. Conversely, the presence of an actual arrhythmia may be associated with anxiety, resulting in misdiagnosis as anxiety or panic disorder [94,95,96]. The study by Rutledge et al. demonstrated that women’s perceptions of their risk for coronary artery disease were more closely linked to symptoms of anxiety than to their actual coronary artery disease status [97]. Moreover, studies have shown an association between anxiety and the severity of symptoms in chronic gastrointestinal diseases [98,99] as well as in functional somatic syndromes [100]. Based on these findings, we hypothesized that the inclusion of trait anxiety into the regression models would contribute to an improvement in their quality. However, no association with symptom intensity was identified. Our negative results may be attributed to the non-uniform relationship between anxiety and symptom perception, as previously demonstrated by Chen et al. [101].

### 4.4. Interoceptive Metrics for Predicting Symptom Score

One of the aims of the current study was to evaluate which of the interoceptive metrics discussed above would result in a better model for predicting symptom scores. Results from simple GLMs showed that (1) the behavioral interoceptive metric IA_MT_, but not IA_HBD_, and (2) the electrophysiological metric ΔHEP_HBD-REST_, but not ΔHEP_MT-REST_, showed associations with symptom score. Association with ΔHEP_HBD-EX_ became not significant after the inclusion of clinical parameters in the model. Despite the correlation between IA_MT_ and ΔHEP_MT-REST_, models including ΔHEP_MT-REST_ showed no associations with symptom score. This may indicate complex interactions between symptom perception, IA, and HEP amplitude. Probably, ΔHEP represents individual perceptive ability, and IA may be just an indicator of this ability. As IA_MT_ did not correlate with ΔHEP_HBD-REST_, we performed a data-driven multivariate regression including these metrics as predictors in a joint regression model, which yielded the best result for predicting symptom score. It was quite unexpected that in binary logistic regression models, HEP_HBD_, but not ΔHEP_HBD-REST_ (as in GLMs), was a significant negative predictor of symptom presence. It can be suggested that for robust prediction (the presence or absence of symptoms), the absolute HEP value during the HBD task is sufficient, but for more accurate prediction (the intensity of symptoms), the HEP modulation (the difference between the resting state and interoceptive task) should be used.

### 4.5. Clinical Applications of Interoception Modulation

Interoception can be manipulated at different levels, through non-invasive stimulation or behavioral approaches [102], offering diverse avenues for enhancing interoceptive abilities. For example, transcutaneous vagus nerve stimulation has been shown to modulate both behavioral [103] and neurophysiological [104] markers of interoception. Similarly, transcranial direct current stimulation over the right or left insula has been demonstrated to significantly improve IA in healthy young individuals [105], whereas stimulation over the somatosensory cortex changed only interoceptive awareness, not IA [106]. Another non-invasive brain stimulation technique, transcranial magnetic stimulation, has demonstrated its ability to alter both IA and HEP amplitudes, suggesting its potential in modifying interoceptive processing [107].

Behavioral approaches, independent of stimulation, also show promise in enhancing interoception. For instance, contemplative practices over a sustained period, such as nine months, have been linked to increased IA and reduced alexithymia scores, as measured by the TAS-20 scale [108].

Additionally, interoceptive training, which uses feedback to improve performance on interoceptive tasks [109,110,111], has been shown to increase IA. The study involving healthy participants revealed that such training can reduce somatic symptom severity (evaluated via the Somatic Perception Questionnaire) and lower state anxiety levels (measured by the STAI questionnaire) [112]. These findings were confirmed in another study with a similar population, which also used functional magnetic resonance imaging to reveal neural circuit changes associated with interoceptive training [23]. Among individuals with somatoform disorders, interoceptive training not only enhanced IA but also led to significant reductions in symptom reporting [110].

Furthermore, interventions targeting interoception have shown efficacy in alleviating symptoms in diverse health conditions. For example, they have been associated with symptom reductions in psychiatric disorders [113], chronic pain conditions [114], and irritable bowel syndrome [115]. Despite these promising findings, research examining the impact of interoceptive interventions among patients with PVCs remains absent. Given the cross-nosological potential of modulating interoception to alleviate symptoms, future studies are essential to explore the therapeutic relevance of behavioral and neurophysiological interoceptive metrics in PVC populations. These investigations could pave the way for novel, evidence-based modulation strategies to address PVC-related symptoms.

## 5. Limitations and Avenues for Further Research

The present work has some limitations that might impact the generalizability of the findings. First, it is based on a modest sample size. Due to the novelty of the study design for these clinical groups, conducting a priori analyses to determine the required sample size for between-group comparisons was not feasible. However, we performed a sample size calculation for within-group analyses, as it was deemed possible to extrapolate data from Schulz et al. (2020) [31], obtained from patients with medically unexplained symptoms of varying severity. Additionally, the reliability of the HEP results profits from the large number of trials per participant (even after the exclusion from the analysis of short trials, eye blink trials, PVCs with two epochs before and one after, and other artifacts). The number of epochs included in each condition per symptomatic and asymptomatic group was comparable with the literature data. Studies reported a similar number of epochs included in each condition of interest, which was 197 ± 22 for a total sample of 16 [116], 182 ± 31 and 169 ± 38 for different conditions for a total sample of 19 [57], and 108 ± 25 and 97 ± 17 for different groups of 25 [117]. Thus, according to a meta-analysis of the relationship between HEP and interoception [3], the average number of participants included in similar studies was 34.8, with 21.8 participants per experimental group. Importantly, previous studies have successfully demonstrated group differences in HEP with sample sizes ranging from 11 to 16 participants (e.g., [33,61,118,119]). Similarly, within-group HEP modulations have been demonstrated in studies with sample sizes comparable to or even smaller than ours (e.g., [116,120,121,122,123]).

Second, there is a significant sex difference between groups, which might bias the interpretations of the psychological questionnaires. With the aim to control for sex-related effects on symptom scores, we used sex as the covariate in multiple regression models. Additionally, moderation analyses for sex did not reveal any significant moderations. Thus, while our data should be considered preliminary, they provide evidence for the existence of a link between interoceptive metrics and symptom severity in PVC populations. Future studies with a larger, more diverse cohort are needed to further investigate the therapeutic relevance of behavioral and neurophysiological interoceptive metrics in this population.

To assess anxiety and alexithymia, we utilized the STAI and TAS-20 questionnaires, which are widely used in studies examining IA, HEP, and psychological traits. The TAS-20 has been shown to be a valid and reliable measure of alexithymia [30] and is widely used in studies exploring interoception in non-psychiatric populations [55,91,108]. Moreover, improvements in IA following contemplative practices were found to be associated with and predictive of changes in emotional awareness as assessed by TAS-20 [108]. Additionally, Santarnecchi et al. observed a correlation between increased right insula thickness and decreased alexithymia levels, also measured with TAS-20 [124]. However, relying on single questionnaires may represent a limitation of this study, potentially reducing the sensitivity of the findings. While the TAS-20 is a well-validated and commonly applied tool, other measures, such as the Levels of Emotional Awareness Scale [125] or the Perth Alexithymia Questionnaire [126], could provide additional insights. Similarly, various instruments exist for assessing anxiety; for instance, a systematic review identified 17 different questionnaires used in related research [127]. This highlights the need for future studies to explore alternative or complementary assessment tools.

An important aspect of our HEP analysis was the exclusion of epochs with PVCs and three epochs time-locked to them [128]. This approach ensured that only basal HEP, which can be considered a marker of interoception as a personal trait, was evaluated. However, it also limited our ability to include patients with a higher PVC burden, which may have hindered the identification of additional associations in our study.

Symptom severity in PVCs can fluctuate over time. Longitudinal studies would provide valuable insights into the relationship between interoception and symptoms.

## 6. Conclusions

To our knowledge, this is the first study to examine the relationship between interoception and symptom severity in PVCs. By applying strict exclusion criteria and selecting only young and middle-aged patients without comorbidities, we aimed to minimize confounding effects, allowing for a clearer analysis of the association between interoception and symptom intensity. Our findings confirm that interoception is significantly associated with PVC symptom severity, supporting our initial hypothesis. The results suggest that integrating both approaches (behavioral and neurophysiological) to assess interoception improves the accuracy of predicting symptom intensity. These findings open promising avenues for the development of novel diagnostic strategies for PVCs, with interoception emerging as a potential target for non-invasive and non-pharmacological treatment approaches.

## Figures and Tables

**Table 1:** Conditions for HEP amplitude comparison between groups.

Condition	Description
1	at rest (HEP_REST_)
2	during the HBD task (HEP_HBD_)
3	during the MT task (HEP_MT_)
4	HEP modulation (ΔHEP) in the HBD task compared to rest (ΔHEP_HBD-REST_)
5	ΔHEP in the MT task compared to rest (ΔHEP_MT-REST_)
6	ΔHEP between the HEP_HBD_ and exteroceptive conditions in the HBD task (ΔHEP_HBD-EX_)

**Table 2:** Clinical parameters of the groups.

	Symptomatic Group, *n* = 17 ^1^	Asymptomatic Group, *n* = 17 ^1^	W/t/χ^2^	*p*
Age, years old	43.0 [39.0; 46.0]	41.0 [38.0; 42.0]	119	0.39 ^a^
Sex, males	3 (18%)	11 (65%)	5.95	**0.015** ^b^
BMI, kg/m^2^	22.5 [20.2; 25.5]	24.6 [23.9; 27]	2.36	**0.025** ^c^
Percent of body fat (%)	26 (24; 31)	25 (19; 32)	−1.02	0.31 ^c^
Smoking	1 (5.9%)	4 (23.5%)	0.94	0.33 ^b^
Pharmacological treatment:	5 (29.4%)	3 (17.7%)	0.16	0.69 ^b^
Beta-blockers	3	0		
Class 1C antiarrhythmics	0	1		
Beta-blockers + Angiotensin II receptor blockers + Calcium channel blockers	0	1		
Beta-blockers + Angiotensin II receptor blockers + Class 1C antiarrhythmics	0	1		
Beta-blockers + Class 1C antiarrhythmics	1	0		
Angiotensin II receptor blockers + Class 1C antiarrhythmics	1	0		
DBP, mmHg.	74 [70; 80]	75.3 [72; 82]	175.5	0.29 ^a^
SBP, mmHg.	106 [102; 116]	110 [105; 118]	0.82	0.42 ^c^
HR, bpm:				
Rest	68.4 [61.8, 74.04]	65.56 [63.3; 72.76]	−0.39	0.6 ^c^
HBD task, interoceptive condition	69.3 [57.5; 73.6]	67.2 [62.1; 73.1]	0.2	0.84 ^c^
HBD task, exteroceptive condition	69.5 [62.4; 75.5]	67.4 [61.8; 72.2]	−0.55	0.59 ^c^
Number of PVCs during experiment:				
Rest	0	10 [0; 20]	202	**0.029** ^a^
HBD task, interoceptive condition	0	3 [0; 7]	196	**0.047** ^a^
HBD task, exteroceptive condition	0	1 [0; 7]	191.5	0.059 ^a^
MT task	0	6 [0; 11]	203.5	**0.015** ^a^
24 h Holter PVCs	3672 [920; 5485]	10,173 [1902; 14,536]	213	**0.018** ^a^
STAI-T (trait scale score)	45 [39; 50]	37 [31; 48]	−1.87	0.07 ^c^
TAS-20-R (total alexithymia index)	43 [36; 48]	35 [29; 39]	−1.57	0.14 ^c^

Note: ^1^ Median and 25th and 75th percentiles—Me [Q1; Q3], *n* (%). ^a^ Wilcoxon nonparametric test for independent samples; ^b^ χ^2^ test; ^c^ Student’s *t*-test. BMI—body mass index, DBP—diastolic blood pressure, CBP—systolic blood pressure, HR—heart rate. Significant *p*-values are in bold.

**Table 3:** Simple (Model A) and multiple (Model B) regression models 3–5.

Model	ΔHEP_CONDITION_	ROI					
	In Predictors	LF	CF	RF	LP	CO	RP
A3	ΔHEP_MT-REST_	0.503	0.766	0.919	0.209	0.124	0.095
B3	ΔHEP_MT-REST_	0.271	0.340	0.904	0.221	0.115	0.124
A4	ΔHEP_HBD-REST_	0.017 ** β = −0.84, SE = 0.35 AIC = 93.92	0.016 ** β = −0.84, SE = 0.35 AIC = 93.13	0.082	**0.037**	0.095	0.103
B4	ΔHEP_HBD-REST_	**0.035**	0.019 * β = −0.98, SE = 0.42 AIC = 87.63	0.094	0.019 * β= −1.05, SE = 0.45 AIC = 88.12	0.018 * β = −0.99 SE = 0.42 AIC = 87.41	**0.033**
A5	ΔHEP_HBD-EX_	0.025 ** β = −0.78, SE = 0.35 AIC = 93.78	0.022 ** β = −0.62, SE = 0.27 AIC = 93.87	0.234	0.153	0.182	0.206
B5	ΔHEP_HBD-EX_	0.131	0.181	0.305	0.466	0.537	0.741

Note: Model A—symptom score ~ predictor, Model B—symptom score ~ predictor + clinical parameters (sex, age, percent body fat, STAI trait scale score, and TAS total alexithymia index). Significant *p*-values are in bold. SE—standard error. ** stands for *p*-values which survived BH correction, * stands for *p* in range 0.05–0.06 after BH correction.

**Table 4:** Final model selection.

Model	ΔHEP_CONDITION_ in Predictors	ROI	Predictors	β	SE	*p*	AIC
A6	ΔHEP_HBD-REST_	LF	IA_MT_ ΔHEP_CONDITION_	0.93 −1.03	0.32 0.38	**0.004** **0.006**	86.05
B6			IA_MT_ ΔHEP_CONDITION_ clinical parameters (male sex)	0.9 −1.12 −2.04	0.37 0.47 0.77	**0.015** **0.016** **0.008**	83.72
A6		CF	IA_MT_ ΔHEP_CONDITION_	0.92 −0.99	0.32 0.36	**0.004** **0.005**	85.14
B6			IA_MT_ ΔHEP_CONDITION_ clinical parameters (male sex)	0.77 −1.04 −2.16	0.34 0.45 0.79	**0.025** **0.022** **0.006**	83.79
A6		LP	IA_MT_ ΔHEP_CONDITION_	1.28 −1.69	0.39 0.52	**0.001** **0.001**	82.49
**B6**			IA_MT_ ΔHEP_CONDITION_ clinical parameters (male sex)	1.11 −1.61 −1.97	0.42 0.56 0.81	**0.008** **0.004** **0.016**	80.86
A7	ΔHEP_HBD-EX_	LF	IA_MT_ ΔHEP_CONDITION_	0.77 −0.67	0.32 0.33	**0.015** **0.044**	88.71
B7			IA_MT_ ΔHEP_CONDITION_ clinical parameters (male sex)	0.71 −0.50 −1.78	0.35 0.44 0.72	**0.039** 0.265 **0.014**	87.95
A7		CF	IA_MT_ ΔHEP_CONDITION_	0.80 −0.55	0.32 0.25	**0.012** **0.029**	88.28
B7			IA_MT_ ΔHEP_CONDITION_ clinical parameters (male sex)	0.74 −0.40 −1.80	0.34 0.36 0.73	**0.031** 0.267 **0.014**	87.95

Note: Model A6–7—symptom score ~ IAMT + ΔHEPCONDITION, Model B6–7—symptom score ~ IAMT + ΔHEPCONDITION+ clinical parameters (sex, age, percent body fat, STAI trait scale score, and TAS total alexithymia index). Significant *p*-values are in bold. For clinical parameters, values for only a significant parameter are indicated (male sex). SE—standard error. AIC—Akaike information criterion.

## Data Availability

The datasets generated and analyzed during the current study and the code we used are openly available in the OSF repository at https://doi.org/10.17605/OSF.IO/BF6P5, accessed on 15 December 2024.
